# Maternal 25(OH)D concentrations ≥40 ng/mL associated with 60% lower preterm birth risk among general obstetrical patients at an urban medical center

**DOI:** 10.1371/journal.pone.0180483

**Published:** 2017-07-24

**Authors:** Sharon L. McDonnell, Keith A. Baggerly, Carole A. Baggerly, Jennifer L. Aliano, Christine B. French, Leo L. Baggerly, Myla D. Ebeling, Charles S. Rittenberg, Christopher G. Goodier, Julio F. Mateus Niño, Rebecca J. Wineland, Roger B. Newman, Bruce W. Hollis, Carol L. Wagner

**Affiliations:** 1 GrassrootsHealth, Encinitas, California, United States of America; 2 Deptartment of Bioinformatics and Computational Biology, The University of Texas MD Anderson Cancer Center, Houston, Texas, United States of America; 3 Medical University of South Carolina Children’s Hospital, Charleston, South Carolina, United States of America; University of Missouri Columbia, UNITED STATES

## Abstract

**Background:**

Given the high rate of preterm birth (PTB) nationwide and data from RCTs demonstrating risk reduction with vitamin D supplementation, the Medical University of South Carolina (MUSC) implemented a new standard of care for pregnant women to receive vitamin D testing and supplementation.

**Objectives:**

To determine if the reported inverse relationship between maternal 25(OH)D and PTB risk could be replicated at MUSC, an urban medical center treating a large, diverse population.

**Methods:**

Medical record data were obtained for pregnant patients aged 18–45 years between September 2015 and December 2016. During this time, a protocol that included 25(OH)D testing at first prenatal visit with recommended follow-up testing was initiated. Free vitamin D supplements were offered and the treatment goal was ≥40 ng/mL. PTB rates (<37 weeks) were calculated, and logistic regression and locally weighted regression (LOESS) were used to explore the association between 25(OH)D and PTB. Subgroup analyses were also conducted.

**Results:**

Among women with a live, singleton birth and at least one 25(OH)D test during pregnancy (N = 1,064), the overall PTB rate was 13%. The LOESS curve showed gestational age rising with increasing 25(OH)D. Women with 25(OH)D ≥40 ng/mL had a 62% lower risk of PTB compared to those <20 ng/mL (p<0.0001). After adjusting for socioeconomic variables, this lower risk remained (OR = 0.41, p = 0.002). Similar decreases in PTB risk were observed for PTB subtypes (spontaneous: 58%, p = 0.02; indicated: 61%, p = 0.006), by race/ethnicity (white: 65%, p = 0.03; non-white: 68%, p = 0.008), and among women with a prior PTB (80%, p = 0.02). Among women with initial 25(OH)D <40 ng/mL, PTB rates were 60% lower for those with ≥40 vs. <40 ng/mL on a follow-up test (p = 0.006); 38% for whites (p = 0.33) and 78% for non-whites (p = 0.01).

**Conclusions:**

Maternal 25(OH)D concentrations ≥40 ng/mL were associated with substantial reduction in PTB risk in a large, diverse population of women.

## Introduction

There were 15 million preterm births (PTB) (<37 weeks) worldwide and more than 1 million infant deaths from PTB complications in 2010 [1]. PTB rates ranged from 5% to 18% around the world and the rate in the United States was disproportionately high (12%) compared to other developed counties [1]. Substantial racial disparities in PTB rates have also been found in the United States: 18% among African Americans, 12% among Hispanics, and 11% among whites in 2009 [2]. Since PTB is the leading cause of neonatal death and multiple short and long term health problems [3], it is critical to identify modifiable maternal risk factors that could significantly reduce PTB risk at the population level.

Multiple epidemiologic studies have found an association between higher maternal serum 25-hydroxyvitamin D [25(OH)D] concentration, the physiological measure of vitamin D status, and lower PTB risk [4–10]. Serum 25(OH)D concentration captures the effect of multiple vitamin D input sources (supplement, sun, and food) and makes provision for inter-individual variability in dose response [11]. Additionally, a *post-hoc* analysis of two pregnancy supplementation trials by Hollis and Wagner et al. found that women with serum 25(OH)D concentrations ≥40 ng/mL had a 59% lower risk of PTB compared to women with concentrations ≤20 ng/mL (p = 0.02) [12]. In those clinical trials, 4000 IU/day of vitamin D was found to safely achieve a concentration of at least 32 ng/mL by early in the second trimester in a diverse group of pregnant women [13–15]. Further, the conversion of 25(OH)D to the active form of the hormone, 1,25(OH)_2_D, was optimized at 40 ng/mL among pregnant women [13].

Based on the demonstrated reductions in PTB in the Hollis and Wagner et al. trials, the Medical University of South Carolina (MUSC) initiated a new standard of care for pregnant women that included routine vitamin D testing and supplementation in September 2015. MUSC is a comprehensive, urban medical center treating a large, diverse population of women (~3000 deliveries/year). The objective of this analysis was to determine if the inverse relationship between maternal 25(OH)D concentration and the rate of PTB found in the earlier randomized controlled trials by Hollis and Wagner et al. could be replicated in the general obstetrical population at MUSC.

## Materials and methods

For this study, following IRB approval (HR#Pro00020570), de-identified medical record data were obtained for all pregnant women aged 18–45 years who received a 25(OH)D test and delivered at MUSC between September 2015 and December 2016. During this time, a protocol was instituted which included routine 25(OH)D testing at each patient’s first prenatal visit. Follow-up testing was recommended between 24–28 weeks and prior to delivery by provider request. Testing increased in May 2016 when the initial 25(OH)D test was automated as part of initial prenatal screening. Total circulating 25(OH)D (ng/mL) was measured using standardized methodology for LC/MS analysis in a MUSC Clinical Laboratory Improvement Amendments (CLIA)-certified clinical laboratory.

Additionally, obstetrical health care providers received education regarding the potential health impact of low vitamin D status via continuing medical education (CME) and nurses provided vitamin D education and standard recommendations for improving vitamin D status using supplements to obstetrical (OB) patients at the initial visit. Vitamin D posters and brochures were available for all patients at MUSC clinics and educational materials were included in the prenatal packet received by all first time OB patients. New OB patients were offered a free bottle of Bio-Tech Pharmacal, Inc. (Fayetteville, AR) vitamin D supplements (5000 IU per capsule) at their first visit and personalized dosing recommendations were provided based on the result of their initial 25(OH)D test using an algorithm of existing dose-response data [11]. The *a priori* goal was the achievement of a maternal 25(OH)D concentration of ≥40 ng/mL to achieve maximal production of the active hormone 1,25(OH)_2_D [13]. Any needed dosing adjustments were made based on follow-up testing.

PTB was defined as delivery of a liveborn infant at <37 weeks gestation. For this analysis, women pregnant with multiples and those participating in vitamin D clinical trials at MUSC were excluded. Chart reviews were conducted to determine whether the PTBs were spontaneous (occurring after preterm labor with intact membranes or preterm pre-labor rupture of the fetal membranes) or indicated. If the PTB was indicated, the reason was also collected.

Maternal serum 25(OH)D concentrations closest to delivery were plotted against gestational age at birth and locally weighted regression (LOESS) was used to explore the relationship in more detail and compare to the Hollis and Wagner et al. trial cohort [12]. PTB rates were calculated for the overall population as well as among women with 25(OH)D concentrations of <20 ng/mL, 20 to <30 ng/mL, 30 to <40 ng/mL and ≥40 ng/mL closest to delivery. These clinically relevant cut points (20 ng/mL from the Institute of Medicine guidelines [16], 30 ng/mL from the Endocrine Society Clinical Practice Guideline [17], and 40 ng/mL from the 25(OH)D concentration found to achieve maximal production of the active hormone 1,25(OH)_2_D [13]) are similar to quartiles based on the distribution of 25(OH)D concentrations (21, 31 and 41 ng/mL). The incidence of PTB across 25(OH)D groups was tested for trend. Logistic regression was used to estimate odds ratios (OR) and 95% confidence intervals for the association between serum 25(OH)D and the risk of PTB. Since socioeconomic status plays a significant role in PTB risk, multivariate logistic regression was used to adjust for socioeconomic status (SES) proxy variables (insurance status and education). Absolute risk reduction (ARR) and number needed to treat (NNT) were also calculated.

To identify differences by race/ethnic group, PTB rates were calculated and logistic regression was conducted for white and non-white women. Additionally, since women with a history of PTB have a known 2 to 3-fold increased risk of a recurrent premature delivery compared to women without a history of PTB [18], logistic regression was conducted for this subgroup as well. While maternal age is also a known risk factor, it was not associated with PTB in univariate analysis within this cohort (p = 0.87).

To assess whether serum 25(OH)D concentrations increased among pregnant patients at MUSC during this time period, first test results and test results closest to delivery were compared using a paired t-test for those with ≥2 tests. The impact of raising 25(OH)D concentrations to ≥40 ng/mL was assessed by comparing PTB rates for women who achieved ≥40 ng/mL on a follow-up test vs. those who did not among women with low 25(OH)D concentrations (<40 ng/mL) on their first test.

Serum calcium concentrations were monitored as clinically indicated. Any cases with identified hypercalcemia (≥11 mg/dL) were reviewed by a member of the Maternal-Fetal Medicine Division at MUSC. Statistical analyses were performed using the R software (www.r-project.org).

## Results

Between September 2015 and December 2016, delivery information was available for 1,064 women with at least one 25(OH)D test result during pregnancy. The characteristics of this cohort are found in Table 1.

**Table 1:** Maternal sociodemographic and clinical characteristics of cohort.

Characteristic	MUSC Cohort
	(N = 1,064)
Race/ethnicity (N, %)*	
White	488 (46%)
African American	395 (37%)
Hispanic	117 (11%)
Asian/PI	19 (2%)
Multiple/Other	39 (4%)
**Maternal age, years (median and range)**	29 (18–45)
**Parity (median and range)**	1 (0–9)
**Gravidity (median and range)**	2 (1–11)
Pre-pregnancy BMI (median and range)*	25 (12–66)
**Marital status (N,%)**	
Married	530 (50%)
**Insurance status (N,%)**	
None/Medicaid	519 (49%)
Education, years (median and range)*	13 (4–20)
**History of Preterm Birth (N,%)**	
Yes	140 (13%)
**Birth Outcome (N,%)**	
Preterm (<37 weeks)	139 (13%)

*6 women missing race/ethnicity data, 31 missing BMI data, and 91 missing education data.

**Fig 1:**
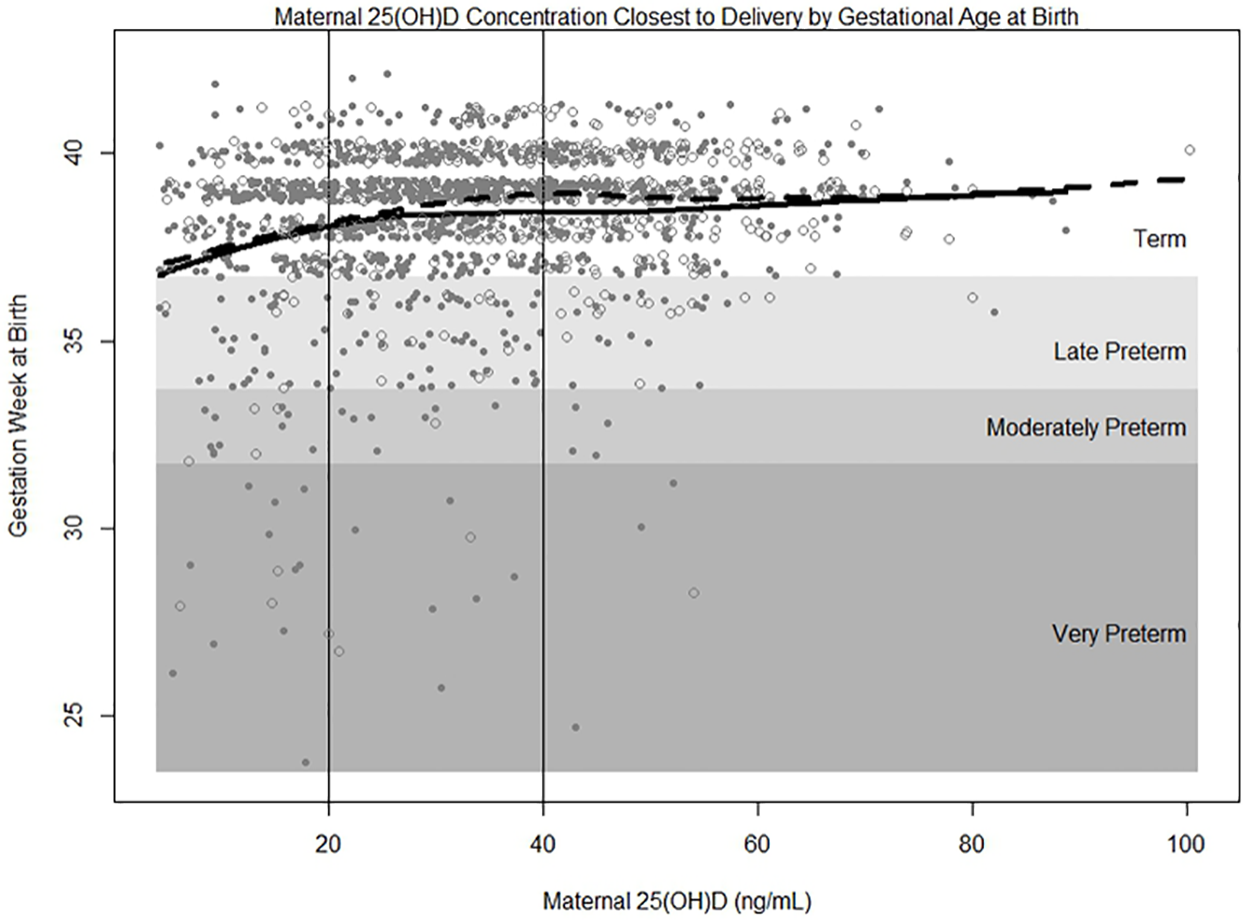
Maternal 25(OH)D concentration by gestational age (weeks) at birth. Term birth is ≥37 weeks of gestation, late preterm birth is 34 to <37 weeks, moderately preterm is 32 to <34 weeks, and very preterm is <32 weeks. Solid gray circles and solid black line represent MUSC cohort (N = 1064) and open circles and dashed line represent Hollis and Wagner et al. trial cohort (N = 509).

**Fig 2:**
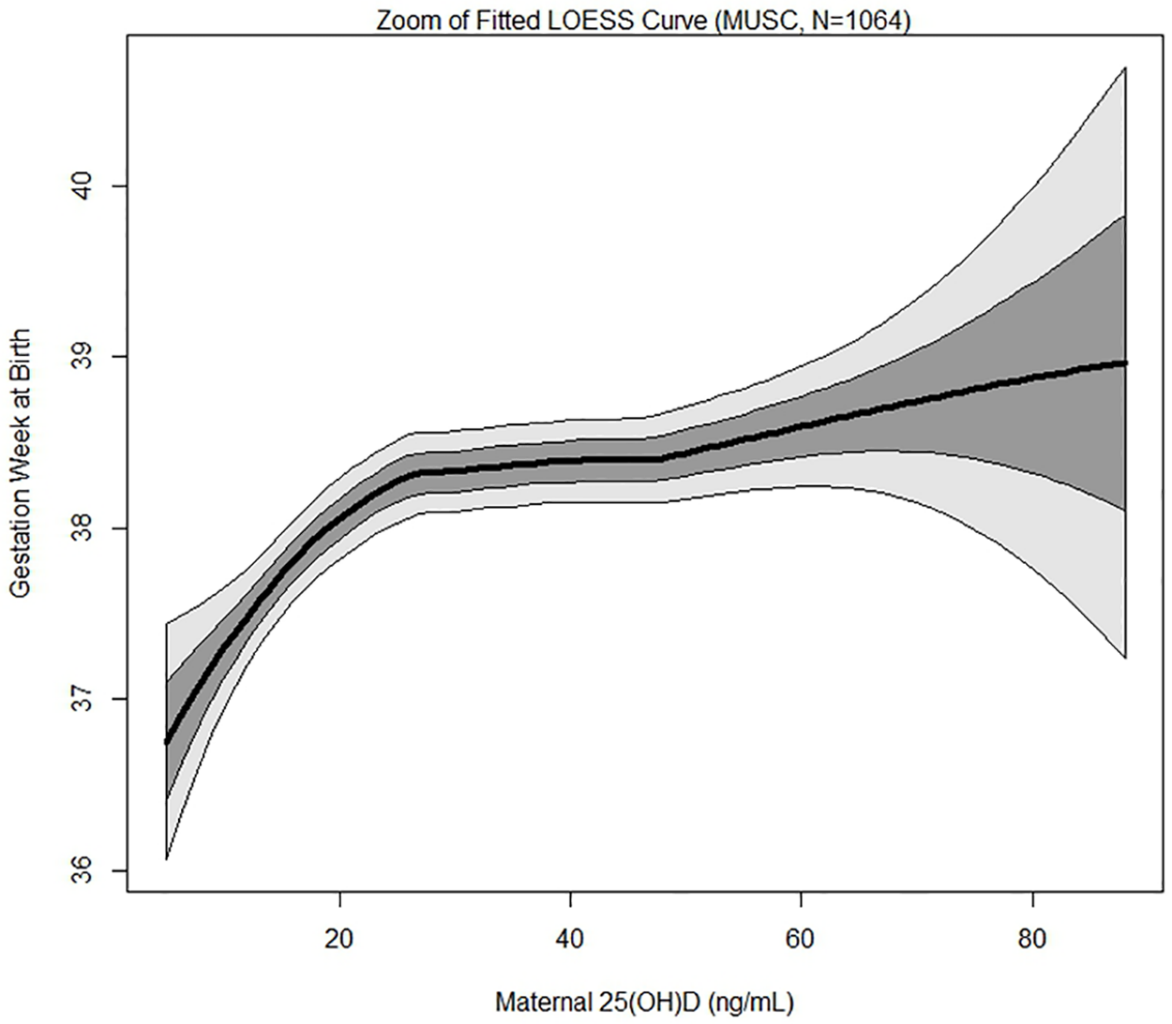
Zoom of the fitted LOESS curve of maternal 25(OH)D concentration and gestational age (weeks) at birth with 1 and 2 SD windows superimposed. Black line represents fitted LOESS curve, dark gray area represents 1 standard deviation, and light gray area represents 2 standard deviations.

There were 139 (13%) PTBs (<37 weeks), of which 20 were very preterm (<32 weeks), 21 were moderately preterm (32 to <34 weeks), and 98 were late preterm (34 to <37 weeks). A plot of gestational age at birth as a function of maternal 25(OH)D concentration with the fitted LOESS curves for both the MUSC cohort and the Hollis and Wagner et al. RCT cohort [12] for comparison is shown in Fig 1. The LOESS curve for the MUSC cohort closely tracks the LOESS curve for the Hollis and Wagner et al. trial cohort. A zoom of the fitted LOESS curve for the MUSC cohort with confidence bounds superimposed shows gestational age at birth initially rising steadily with increasing maternal 25(OH)D concentration and then reaching a plateau at approximately 30–50 ng/mL (Fig 2).

PTB rates were 20% in women with 25(OH)D <20 ng/mL (N = 248), 12% in women with 25(OH)D 20 to <30 ng/mL (N = 267), 13% in women with 25(OH)D 30 to <40 ng/mL (N = 255) and 9% in women with 25(OH)D ≥40 ng/mL (N = 294) (Table 2). Those with serum 25(OH)D concentrations ≥40 ng/mL had a 62% lower risk of PTB compared to those with concentrations <20 ng/mL (OR = 0.38, 95% CI = 0.23–0.63, p<0.0001; AAR = 11.3%; NNT = 9) (Table 2). There was a similar lower risk (59%) after adjusting for SES variables (insurance status and education) (OR = 0.41, 95% CI = 0.24–0.72, p = 0.002).

**Table 2:** Association between maternal 25(OH)D concentration and the risk of preterm birth (N = 1064).

	Preterm Birth (<37 Weeks)	Term Birth (≥37 Weeks)	p-value (test for trend)	OR	SES Adjusted† OR
				(95% CI)	(95% CI)
<20 ng/mL	49 (20%)	199 (80%)	**0.0003**	1.0	1.0
N (%)					
20 to <30 ng/mL	33 (12%)	234 (88%)		**0.57**	0.63 (0.37,1.04)
N (%)				**(0.35, 0.93)**	
30 to <40 ng/mL	32 (13%)	223 (87%)		**0.58**	**0.53 (0.31,0.91)**
N (%)				**(0.36, 0.95)**	
≥40 ng/mL	25 (9%)	269 (91%)		**0.38**	**0.41 (0.24,0.72)**
N (%)				**(0.23, 0.63)**	

Bold values signify significance at p<0.05.

^†^Adjusted for insurance status and years of education (social economic status proxy variables).

Of the 139 PTBs, approximately half were spontaneous (47%) and half were indicated (53%), of the latter, 66% were indicated for maternal hypertensive disorders. PTB rates by serum 25(OH)D group and PTB subtype are shown in Table 3. For those with 25(OH)D concentrations ≥40 ng/mL compared to those with 25(OH)D concentrations <20 ng/mL, there was a 58% lower risk of spontaneous PTB (OR = 0.42, 95% CI = 0.20–0.89, p = 0.02; AAR = 4.8%; NNT = 21) and a 61% lower risk of indicated PTB (OR = 0.39, 95% CI = 0.20–0.76, p = 0.006; AAR = 6.5%; NNT = 15) (Table 3).

**Table 3:** Association between maternal 25(OH)D concentration and the risk of preterm birth by preterm birth subtype.

	Spontaneous Preterm Birth Rates	Spontaneous Preterm Birth: OR	Indicated Preterm Birth Rates	Indicated Preterm Birth: OR
	(<37 Weeks)	(95% CI)	(<37 Weeks)	(95% CI)
<20 ng/mL (N = 248)	21 (8%)	1.0	28 (11%)	1.0
N preterm (%)				
20 to <30 ng/mL (N = 267)	17 (6%)	0.74	16 (6%)	**0.50**
N preterm (%)		(0.38, 1.43)		**(0.26, 0.95)**
30 to <40 ng/mL (N = 255)	16 (6%)	0.72	16 (6%)	**0.53**
N preterm (%)		(0.37, 1.42)		**(0.28, 0.99)**
≥40 ng/mL (N = 294)	11 (4%)	**0.42**	14 (5%)	**0.39**
N preterm (%)		**(0.20, 0.89)**		**(0.20, 0.76)**

Bold values signify significance at p<0.05.

The frequency of PTB was 10% among white women and 15% among non-white women. African American women accounted for a majority (69%) of the non-white births and had a 19% PTB rate. There was a 65% lower risk of PTB among white women with 25(OH)D ≥40 ng/mL compared to those <20 ng/mL (OR = 0.35, 95% CI = 0.13–0.92, p = 0.03; AAR = 13.8%; NNT = 7) and a 68% lower risk among non-white women (OR = 0.32, 95% CI = 0.14–0.74, p = 0.008; AAR = 11.7%; NNT = 9).

**Fig 3:**
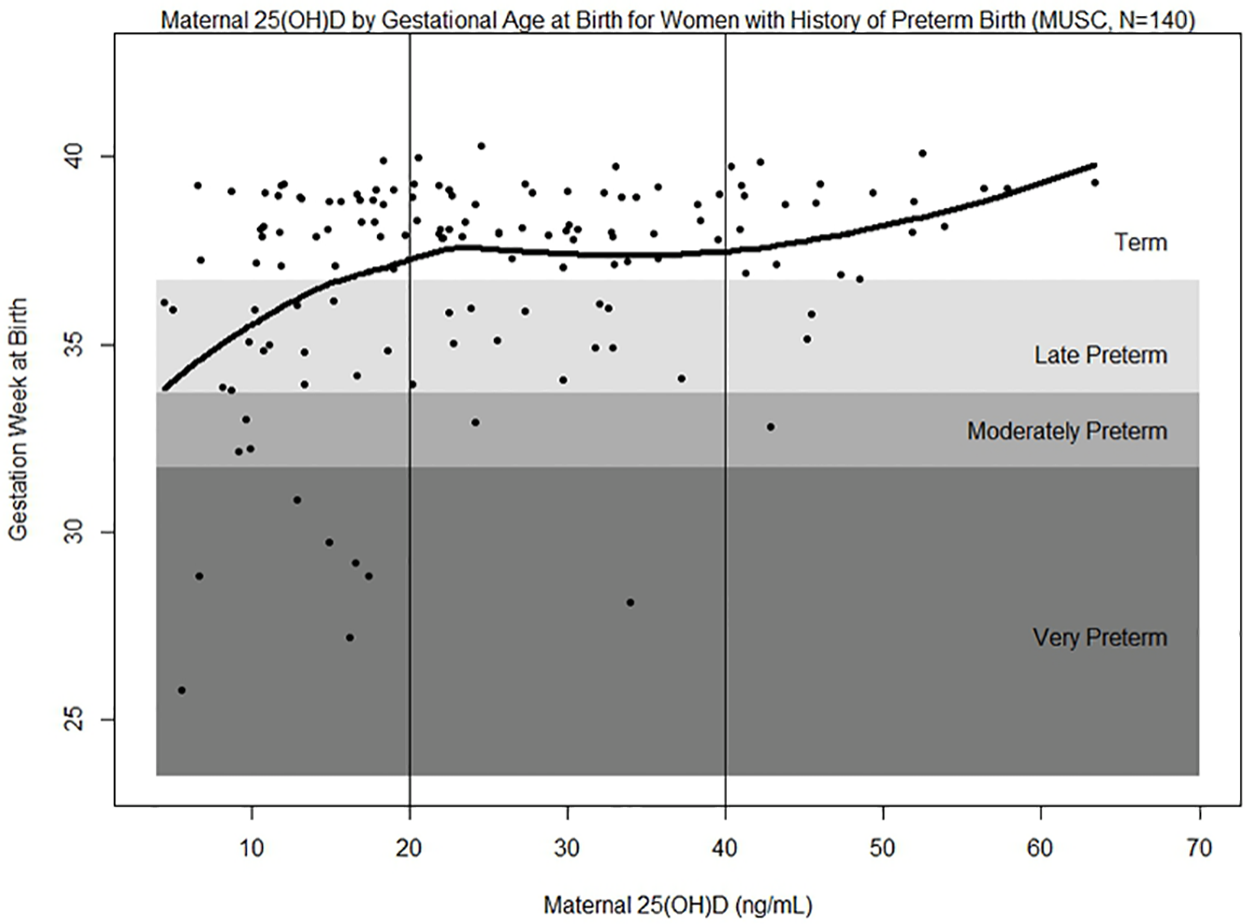
Maternal 25(OH)D concentration by gestational age (weeks) at birth for women with a prior preterm birth. Term birth is ≥37 weeks of gestation, late preterm birth is 34 to <37 weeks, moderately preterm is 32 to <34 weeks, and very preterm is <32 weeks. Black line represents fitted LOESS curve.

Since having had a previous PTB is a significant risk factor for a recurrent PTB, we also assessed the relationship between PTB and 25(OH)D for these women separately. Among the 140 women (13% of cohort) with a prior PTB, 29% experienced a recurrent PTB. A plot of gestational age at birth as a function of maternal 25(OH)D concentration with the fitted LOESS curve for women with a history of PTB is shown in Fig 3. Recurrent PTB rates for this subgroup were 42% in women with 25(OH)D <20 ng/mL (N = 57), 24% in women with 25(OH)D 20 to <30 ng/mL (N = 34), 24% in women with 25(OH)D 30 to <40 ng/mL (N = 25) and 13% in women with 25(OH)D ≥40 ng/mL (N = 24). There was an 80% lower risk of recurrent PTB with 25(OH)D ≥40 ng/mL compared to those <20 ng/mL among women with a prior PTB (OR = 0.20, 95% CI = 0.05–0.74, p = 0.02; AAR = 29.6%; NNT = 3).

**Fig 4:**
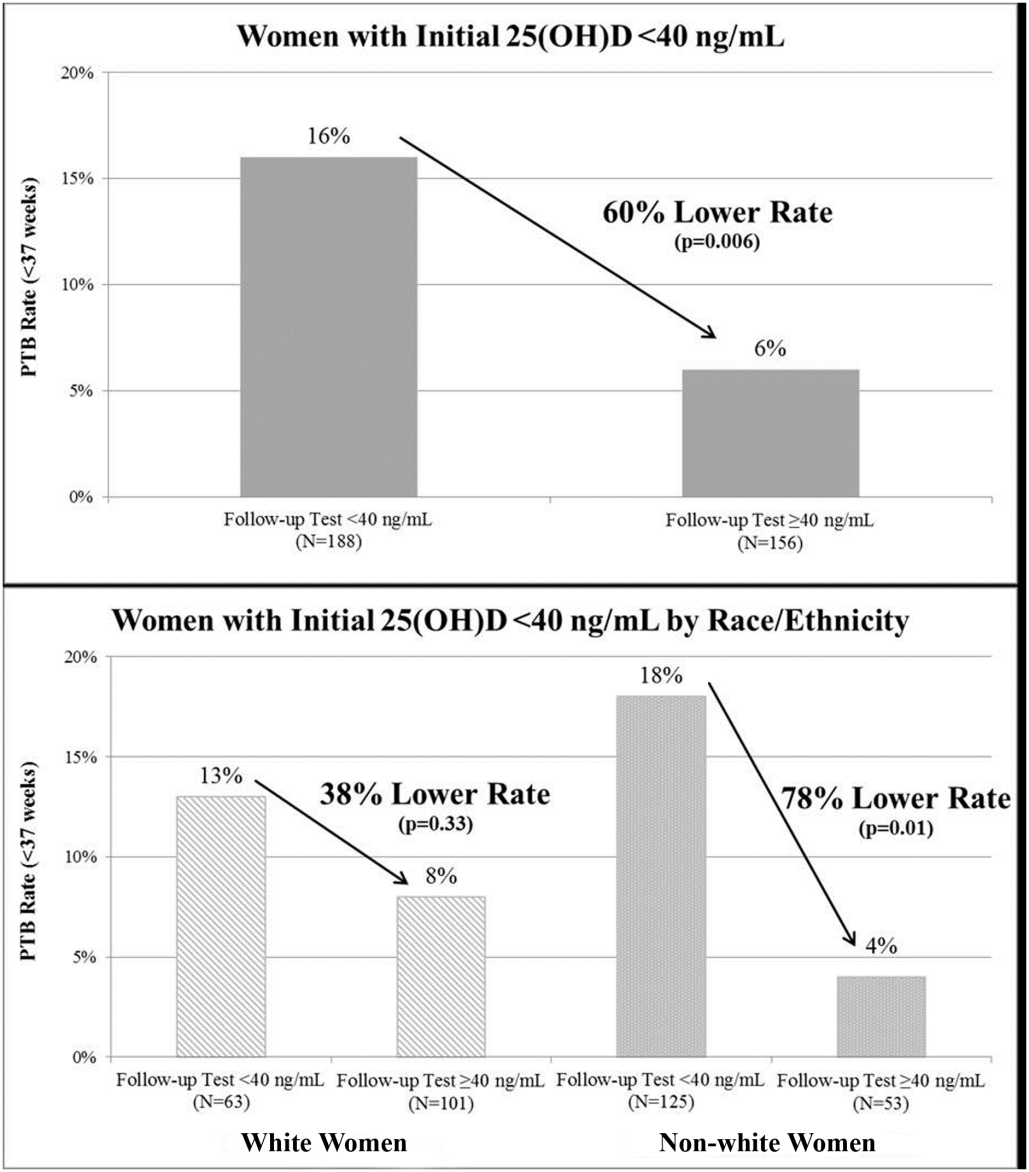
Preterm birth rates among women with low initial 25(OH)D concentrations (<40 ng/mL at ≤20 weeks), comparing those with concentrations ≥40 ng/mL vs. those with concentrations <40 ng/mL on a follow-up test.

Among women with ≥2 tests (N = 390), there was an increase in mean 25(OH)D concentration from 24 ng/mL to 39 ng/mL (p<0.0001). For those with ≥2 measurements and initial 25(OH)D concentrations <40 ng/mL at 20 weeks or earlier (N = 344), the PTB rate was 60% (95% CI = 20–80%, p = 0.006) lower for those who achieved ≥40 ng/mL on a follow-up test compared to those who did not raise their 25(OH)D concentrations to the target concentration of ≥40 ng/mL on a follow-up test (6% and 16% respectively, AAR = 9.6%; NNT = 10) (Fig 4). By race/ethnicity, the PTB rate was 38% (95% CI = -58-75%, p = 0.33) lower among white women (8% and 13% respectively, AAR = 4.8%, NNT = 21) and 78% (95% CI = 12–95%, p = 0.01) lower among non-white women (4% and 18% respectively, AAR = 13.8%, NNT = 7). There were no cases of vitamin D toxicity based on elevated serum calcium levels (≥11 mg/dL).

## Discussion

We found a clear association between maternal 25(OH)D concentration and PTB risk in the general obstetrical population at an urban medical center treating a large, diverse population of women. Women with a 25(OH)D concentration of ≥40 ng/mL had a 62% lower risk of PTB compared to those with concentrations <20 ng/mL. Similar or greater magnitudes of decreased risk were observed for PTB subtypes, race/ethnic groups, and among women with a prior PTB. Additionally, among women with low initial 25(OH)D concentrations (<40 ng/mL), PTB rates were significantly lower (60%) for those who achieved ≥40 ng/mL on a follow-up test vs. those who did not, especially for non-white women (78%).

These findings support the inverse association between maternal 25(OH)D and PTB risk found in the Hollis and Wagner et al. randomized clinical trials [12,14,15] as well as epidemiological studies [4–10]. Wagner et al. found a 59% lower risk of PTB for women with serum 25(OH)D concentrations ≥40 ng/mL compared to those with concentrations ≤20 ng/mL (p = 0.02) [12]. Bodnar et al. found a 56% lower risk of PTB among those with 25(OH)D ≥30 ng/mL compared to <20 ng/mL (95% CI = 23–62%) [4]. A 70% lower risk of PTB was found for women ≥20 ng/mL vs. <20 ng/mL in a study by Perez-Ferre et al. (p = 0.002) [5]. Two recent meta-analyses also found that higher 25(OH)D concentrations significantly decreased the risk of PTB [9,10]. *Post-hoc* analysis of the Hollis and Wagner et al. trials [12] and the Bodnar et al. study [4] assessed the non-linear relationship between 25(OH)D and PTB, both identifying a decreasing risk of PTB as 25(OH)D increased to approximately 40 ng/mL. Fig 1 shows that the increase in gestational age at birth with rising 25(OH)D concentrations is similar between the general obstetrical population at MUSC and the Hollis and Wagner et al. RCT cohorts [12].

Similar magnitudes of decreased risk for women with 25(OH)D concentrations ≥40 ng/mL vs. <20 ng/mL were observed among PTB subtypes: spontaneous PTB (58%) and indicated PTB (61%). Bodnar et al. also found similar reductions in PTB risk for spontaneous and medically indicated PTBs [4]. These findings show that higher vitamin D status is significantly associated with lower PTB risk for both PTB subtypes and that vitamin D plays an important role in the underlying causes of indicated PTB including maternal hypertension, pre-existing diabetes, and non-reassuring fetal status. Improving the vitamin D status of women with underlying conditions may avert the need for a medically indicated preterm delivery.

Subgroup analyses revealed some potentially key findings. The association between 25(OH)D and reduced risk of PTB was consistent across race/ethnic groups. There were significant decreases in PTB risk for ≥40 ng/mL vs. <20 ng/mL for both white (65%) and non-white women (68%). These findings suggest that improvements in vitamin D status may decrease the disparity in PTB rates between race/ethnic groups. Additionally, for women with a prior PTB, the substantial decrease in recurrent PTB risk (80%) for ≥40 ng/mL vs. <20 ng/mL indicates that improving vitamin D status in this high risk subgroup could sizably reduce PTB rates.

Among women with initial 25(OH)D concentrations <40 ng/mL, the significantly lower PTB rate (60%) for those who achieved ≥40 ng/mL compared to those who did not reach the target concentration on a follow-up test suggests that maternal vitamin D status is a modifiable risk factor that can be addressed during the prenatal period. Especially notable is the considerably lower PTB rate for those who reached ≥40 ng/mL vs. those who did not (78%) among non-white women. This indicates that improving the vitamin D status of non-white women, who are known to have particularly low vitamin D concentrations, could dramatically decrease racial disparities in PTB rates. These findings are supported by the Hollis and Wagner et al. randomized controlled trials which found that among women with low baseline 25(OH)D concentrations (≤20 ng/mL), there was a lower risk of PTB (78%) for those who achieved ≥40 ng/mL compared to those who did not (RR = 0.22, 95% CI = 0.05–0.92) [12].

Strengths of this analysis include assessing the relationship between maternal 25(OH)D and PTB risk in the general obstetrical population using medical record data at an urban medical center treating a large, diverse population of women. This overcomes the bias of subject selection and allowed a large sample that was more representative of the larger obstetrical population since it included a diversity of socioeconomic backgrounds, race/ethnic groups, pregnancy-related conditions, and medical histories. A larger sample size also enabled subgroup analysis by PTB subtype, race/ethnicity, and among women with a previous PTB. Additionally, using serum 25(OH)D concentration is a better indicator of vitamin D status and statistically more powerful than using reported intake because it accounts for all vitamin D input sources (sun exposure, food, and supplements) and makes provision for inter-individual variability in vitamin D dose response.

A limitation of this study was that nearly two-thirds of the women (63%) had only one 25(OH)D test during pregnancy, which prohibited assessment by trimester and a more thorough analysis of vitamin D benefit among those with low initial levels. This is due to follow-up testing being on the recommended test order set but still needing provider selection compared to the initial test which is automated as part of the initial prenatal screening. Additionally, the initial test was not automated until May 2016 so many women who delivered during the early study time period did not have a 25(OH)D measurement and therefore could not be included in this analysis. Since this new standard of care was implemented in September 2015, there have been ongoing improvements in automation, provider practice, and integration within the existing MUSC health care system. Further evaluation of the vitamin D testing and supplementation protocol is planned.

Vitamin D status is a key modifiable maternal risk factor for the prevention of PTB. The findings from this analysis support the previously identified association between higher maternal 25(OH)D concentrations and reduced risk of PTB and shows that the benefits identified in the RCTs by Hollis and Wagner et al. are achievable in a general obstetrical population. These findings also highlight the importance of achieving a 25(OH)D concentration substantially above 20 ng/mL, the concentration recommended by the IOM for pregnant women [16], for PTB prevention. Having a 25(OH)D concentration of at least 40 ng/mL during pregnancy could reduce PTB risk by >60% and would achieve the optimal conversion of 25(OH)D to 1,25(OH)_2_D [13]. Vitamin D testing and supplementation of pregnant women is a safe, affordable prevention tool that could substantially reduce the occurrence of PTB and the heavy burden of associated morbidity, mortality and economic costs.
